# Retraction: Population growth poses a significant threat to forest ecosystems: A case study from the Hindukush-Himalayas of Pakistan

**DOI:** 10.1371/journal.pone.0343943

**Published:** 2026-03-03

**Authors:** The PLOS One Editors

After this article [1] was published, concerns were raised regarding inconsistencies in the reporting of the forest cover results. Specifically:

- The forest cover results differ between Fig 1, Fig 2, Table 3, and subsections 3.1 “Forest cover maps” and 3.2 “Forest cover change” of the Results section.
- In the Table 3 October 1980 to 2021 and Fig 2 October 1980 to 2020 results, the deforestation percentage result does not appear to correspond to the reported time period and forest cover results.
- Differences were noted in the reporting of the time periods and collection years for the forest cover data. Table 3 reports October 2000 to 2021, Fig 2 reports October 2000 to 2020, and Fig 4 reports 2001–2020.

Co-corresponding author NA stated that the results in Fig 1 and subsection 3.1 “Forest cover maps” represent the area of the largest single forest patch for each respective year and do not show the total forest cover, whilst Table 3 and subsection 3.2 “Forest cover change” show total forest cover for the entire study area.

Regarding the Table 3 October 1980 to 2021 and Fig 2 October 1980 to 2020 results, co-corresponding author NA stated the deforestation area of 6509 ha and the deforestation percentage of 1.4% are incorrect and should instead be 7109 ha and 1.18%, and that the Fig 2 October 2000 to 2020 deforestation result of 4265 ha should instead be 4965 ha.

Regarding the differences in reported time periods, co-corresponding author NA stated that in Table 3, “October 2000 to 2021” and “October 1980 to 2021” are incorrect and should instead read “October 2000 to 2020” and “October 1980 to 2020”. The authors did not comment on the 2001–2020 time period reported in Fig 4.

Co-corresponding author NA stated that in subsection 3.4 “Decay model forecast” of the Results section in [1], the population density is incorrectly written as “0.28 million in 1851” and should instead read that the population density was 0.28 million in 1951.

In light of the extent of the above concerns, which bring into question the reliability of the reported results, the *PLOS One* Editors retract this article.

NA did not agree with the retraction. ZU, BA, AA, and KS either did not respond directly or could not be reached.

In addition to the above concerns, the *PLOS One* Editors identified multiple citation errors in this article [1].
